# Pathophysiological frameworks and emerging preventative strategies in SUDEP

**DOI:** 10.3389/fneur.2026.1916085

**Published:** 2026-08-10

**Authors:** Chenxi Xu, Yiyuan Wang, Suping Nie, Jia Gao, Yunxiang Guan

**Affiliations:** 1 First Clinical Medical College of Henan University of Chinese Medicine, Zhengzhou, Henan, China; 2 Department of Encephalopathy, The First Affiliated Hospital of Henan University of Traditional Chinese Medicine, Zhengzhou, Henan, China

**Keywords:** biomarkers, central autonomic network, neurostimulation, pathophysiology, prevention, SUDEP

## Abstract

Sudden Unexpected Death in Epilepsy (SUDEP) represents a leading cause of epilepsy-related mortality, reflecting a severe disruption of systemic physiology that remains challenging to address using standard clinical protocols. Although generalized tonic–clonic seizures (GTCS) and pharmacoresistance are established risk indicators, current clinical risk stratification frequently lacks the predictive granularity required to anticipate individual cardiorespiratory collapse. This review provides a comprehensive synthesis of the SUDEP pathophysiological framework, aiming to bridge the gap between retrospective clinical observation and proactive precision medicine. We delineate a translational framework focused on the intrinsic biological susceptibility of the neuro-cardiac axis, particularly the genetic “dual pathology” of channelopathies and structural cardiac remodeling. Furthermore, we evaluate the transition from reactive risk identification toward multimodal “digital phenotyping” leveraging artificial intelligence. Finally, we assess the developmental trajectory of “secondary prevention” strategies, such as automated neurostimulation, designed to interrupt the terminal cascade. By integrating genomic vulnerability with emerging preventative technologies, this review outlines a multidisciplinary approach to transforming SUDEP into a more predictable and preventable clinical entity.

## 1 Introduction

Epilepsy remains a pervasive chronic neurological disorder, affecting approximately 6.38 per 1,000 individuals globally (1). Among its complications, Sudden Unexpected Death in Epilepsy (SUDEP) stands as the most severe, accounting for up to 18% of epilepsy-related mortalities (56). Strictly defined as a sudden, non-traumatic, and non-drowning death in an individual with epilepsy, excluding status epilepticus and toxicological causes, SUDEP represents a critical and often terminal failure of systemic physiology (2, 3). Despite being clinically recognized for over a century, since its early description in The Lancet in 1868, the definitive etiology has remained elusive, leaving clinicians with limited tools for effective prediction and prevention. Current pathophysiological hypotheses converge on the theory of a “perfect storm,” a multifaceted cascade involving respiratory arrest, cardiac arrhythmia, and global cerebral suppression (4, 5). While epidemiological data have robustly identified generalized tonic–clonic seizures (GTCS) as the primary precipitating factor, risk stratification based solely on seizure frequency has proven insufficient for precise individual forecasting. A critical gap remains in understanding why similar seizure burdens lead to physiological recovery in some patients but progress to fatal outcomes in others. This inherent unpredictability highlights the urgent need to move beyond retrospective clinical inventories and identify objective, quantifiable biomarkers that can signal an impending breakdown in autonomic homeostasis.

While recent methodologically rigorous prospective cohorts, such as the multicenter analyses by Ochoa-Urrea et al. (6) and the REPO2MSE study (7), which have significantly advanced the understanding of epidemiological risks and peri-ictal electrographic markers, an opportunity exists to complement these population-level findings with individual-level translational models. Large-scale clinical inventories are essential for assessing statistical risk but may not fully outline proactive engineering solutions for individual patients. Therefore, this review proposes a conceptual framework that emphasizes forward-looking precision medicine. We synthesize the intrinsic biological susceptibility of the neuro-cardiac axis, specifically genetic “dual pathology” and structural autonomic remodeling—with emerging preventative technologies. By evaluating the transition toward AI-assisted “digital phenotyping” and the ongoing research into closed-loop neurostimulation, this review aims to provide a translational roadmap for the active prevention of SUDEP.

## 2 Epidemiology

The incidence of SUDEP is highly variable, contingent upon the population studied and the rigor of surveillance. In the general epilepsy population, incidence rates are estimated at 1.2 per 1,000 person-years (PY) (8). However, this risk is not uniformly distributed. In cohorts with refractory epilepsy or surgical candidates, rates escalate dramatically to 6.3–9.3 per 1,000 PY, compared to 0.35–2.3 in community-based samples (9). The cumulative lifetime risk can reach up to 35% (61), posing a substantial threat over a patient’s lifespan. Age-dependent vulnerability is a critical feature. While historically considered lower in young children (0.22/1,000 PY), recent data suggest that adolescents (>12 years) face risks comparable to adults. Furthermore, socioeconomic disparities exacerbate this burden; mortality is disproportionately higher in low- and middle-income countries (LMICs) due to limited access to antiseizure medications (ASMs) and specialized care (10).

## 3 Pathophysiologic mechanisms: the “perfect storm” theory

**Figure 1:**
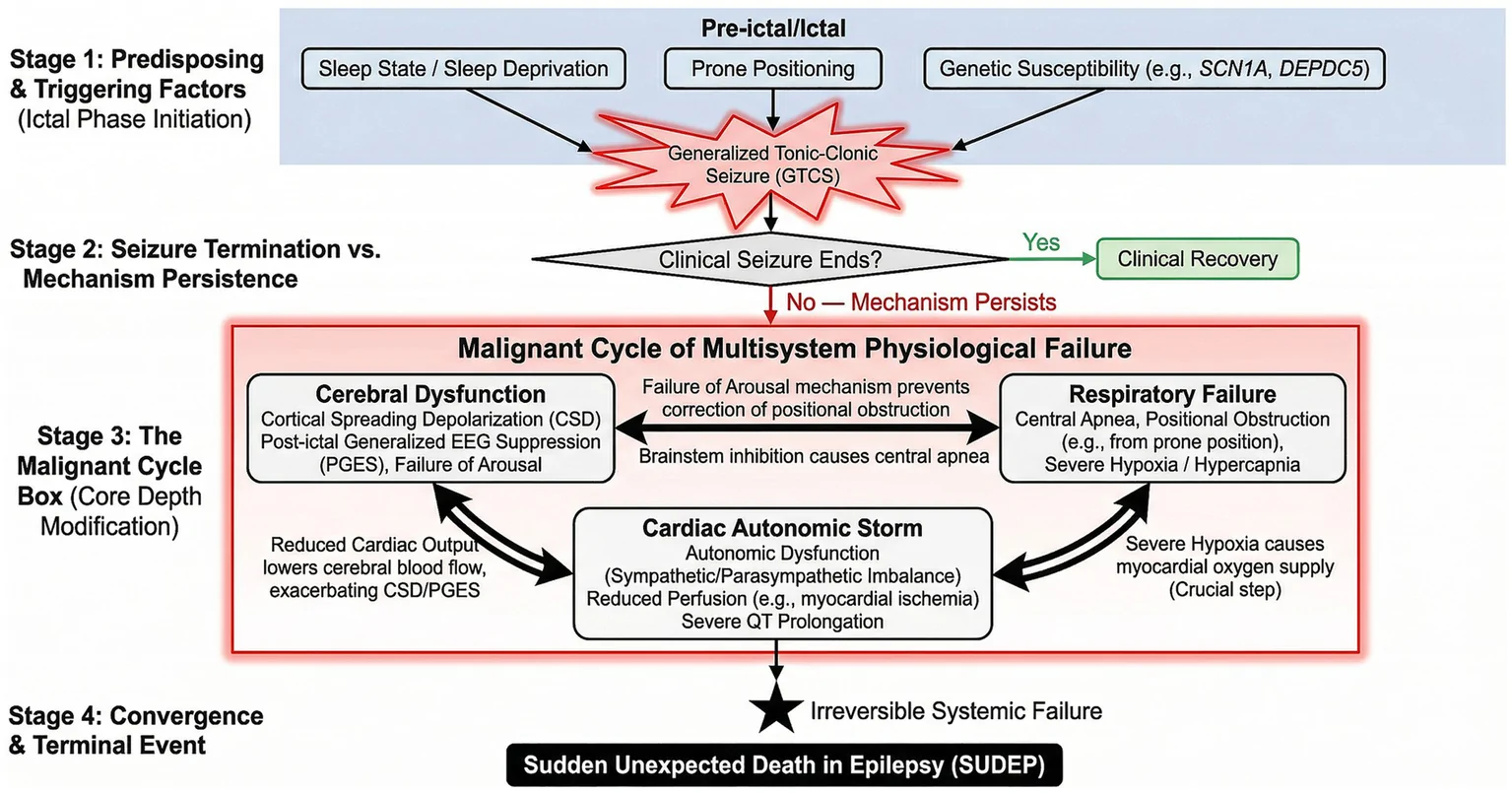
The ‘Perfect Storm’ of SUDEP: a malignant cycle of multisystem failure.

Preclinical models and clinical case series suggest that the specific mechanisms underlying SUDEP are multifactorial and highly interconnected (59). SUDEP is associated with a multi-system cascade of abnormalities affecting cardiac, respiratory, serotonergic, autonomic and cerebral function (4, 5). Figure 1 conceptualizes SUDEP not as a sudden post-ictal accident, but as a dynamic ictal-to-post-ictal continuum. While the clinical collapse often peaks after seizure termination, the terminal cascade is frequently initiated during the ictal phase as hypersynchronous activity propagates from the cortex to subcortical autonomic centers, including the brainstem. This subcortical propagation can trigger early respiratory dysfunction or cardiac instability long before the seizure clinically concludes. As highlighted by Singh et al. (5), the multisystem nature of this failure suggests that the “perfect storm” is a heterogeneous process that does not strictly adhere to seizure phases.

### 3.1 Unraveling post-ictal central respiratory depression: beyond the serotonergic hypothesis

Post-ictal central respiratory dysfunction represents a critical pathophysiological driver of SUDEP. While the landmark MORTEMUS study robustly established centrally mediated terminal apnea as the primary event in the majority of observed SUDEP cases, interpreting this apnea as a singular, uniform mechanism oversimplifies a highly complex failure of respiratory control. Contemporary basic and translational research indicates that this respiratory collapse is a multifactorial process, extending far beyond the traditional serotonergic paradigms. Historically, the serotonergic (5-HT) hypothesis has dominated the discourse on SUDEP-related respiratory failure (11). Preclinical models, particularly DBA/1 and DBA/2 mice, have demonstrated that seizure-induced spreading depolarization into the brainstem compromises the medullary raphe nuclei (12). This targeted depletion of 5-HT signaling blunts the hypercapnic ventilatory response (HCVR) (56). Consequently, when post-ictal hypoxemia and hypercapnia naturally occur, the impaired 5-HT network fails to trigger the vital physiological arousal required to clear the airway, trapping the patient in an irreversible cycle of central depression.

However, recent neurobiological investigations emphasize that respiratory abnormalities in SUDEP involve a broader array of neuromodulatory and network-level dysfunctions. Chief among these is the purinergic system. During severe generalized tonic–clonic seizures (GTCS), extreme neuronal firing and ATP hydrolysis lead to a substantial extracellular accumulation of adenosine. This “purinergic overdrive” exerts profound inhibitory effects via adenosine A1 receptors located in the brainstem’s preBötzinger complex—the primary generator of respiratory rhythm. Preclinical studies have shown that administering systemic or local adenosine antagonists (e.g., caffeine or specific A1/A2A receptor blockers) can successfully prevent seizure-induced respiratory arrest, highlighting adenosine as an equally critical, non-serotonergic mediator of terminal apnea (13, 14). Furthermore, human intracranial electroencephalography (iEEG) studies have unveiled a critical network-level mechanism originating in the limbic system. Direct electrical stimulation of the amygdala has been shown to elicit central apnea. Crucially, these patients report a complete absence of dyspnea (air hunger); they are subjectively unaware that they have stopped breathing (15). This phenomenon suggests that seizure propagation into the amygdala not only suppresses the brainstem respiratory drive but also simultaneously abolishes the perceptual warning signals of hypoxia, perfectly illustrating why patients fail to awaken or change positions post-ictally.

Therefore, the respiratory phase of the “perfect storm” is a synergistic, multi-network failure. While specific subcortical involvement, such as seizure propagation into the amygdala, has been shown in focal models to directly halt breathing and dangerously abolish the perceptual warning signals of hypoxia (air hunger), the definitive respiratory arrest observed during a widespread bilateral tonic–clonic seizure (BTCS) cannot be attributed to a single structure. Instead, it reflects a massive, simultaneous disruption across numerous cortical and subcortical brain networks. During this global bioelectric storm, the initial amygdalar and limbic suppression of respiratory drive is rapidly compounded by the subsequent adenosine surge and 5-HT depletion, which ultimately paralyze the medullary pacemakers and abrogate chemical arousal reflexes.

### 3.2 Cardiac mechanisms: brain-heart interactions, autonomic dysfunction, and the “epileptic heart”

While central respiratory failure is frequently the initiating domino in the terminal cascade, cardiac instability acts as a critical and independent co-conspirator. Historically, cardiac arrhythmias in SUDEP were viewed simply as secondary consequences of hypoxia (16). However, contemporary electrophysiological investigations reveal a highly complex, bidirectional “brain-heart interaction.” Seizures can induce a wide variety of transient cardiac effects during the peri-ictal phase, including profound alterations in blood pressure, heart rate variability (HRV), complex arrhythmias, asystole, and other dynamic ECG abnormalities (17). In severe cases, the intense peri-ictal physiological stress can even trigger the development of Takotsubo syndrome (18).

This acute arrhythmogenic vulnerability is driven by profound dysregulation of the autonomic nervous system. The brainstem, which hosts critical parasympathetic nuclei such as the dorsal motor nucleus of the vagus and the nucleus ambiguus, plays a central role in mediating autonomic cardiac reflexes (19). During a generalized tonic–clonic seizure (GTCS), hypersynchronous discharges propagating into cortical areas, such as the posterior insula and cingulate cortex, as well as subcortical structures like the amygdala and hypothalamus, fundamentally disrupt this delicate autonomic balance (20). This disruption can cause an acute sympathetic storm, clinically manifested as severe ictal tachycardia, followed by rapid, paradoxical parasympathetic (vagal) overcompensation. This fluctuating autonomic tone severely impairs baroreflex sensitivity, predisposing the patient to life-threatening arrhythmias and profound repolarization instability long before critical hypoxia sets in.

Beyond these acute functional shifts, the inherent vulnerability of the cardiovascular system is exacerbated by chronic structural changes, recently formalized as the concept of the “epileptic heart.” This paradigm defines the epileptic heart as the cumulative damage to the myocardium and coronary vasculature caused by chronic epilepsy. Repeated seizure-induced surges in catecholamines and recurrent hypoxemia lead to progressive electrical and mechanical dysfunction, often manifesting as myocardial fibrosis and structural cardiomyopathies (21). Consequently, individuals with epilepsy exhibit a significantly higher rate of these specific cardiovascular comorbidities, which drastically lowers the threshold for ventricular fibrillation during a bioelectric storm.

### 3.3 Cerebral dysfunction, cortical spreading depolarization, and the failure of arousal

Cerebral dysfunction constitutes the terminal link in the SUDEP cascade, primarily manifesting as a profound failure of the arousal mechanisms necessary to restore systemic homeostasis (22). While earlier models often simplified this as generalized “post-ictal coma,” advanced electrophysiological and preclinical studies reveal a highly specific, multiphasic shutdown of cortical and brainstem networks.

At the cellular level, this widespread cerebral inhibition is increasingly attributed to Cortical Spreading Depolarization (CSD). CSD is not merely a cessation of electrical activity; original live-imaging and multielectrode array studies in rodent models have defined it as a slowly propagating wave of near-complete breakdown in transmembrane ion gradients. During a severe seizure, the massive efflux of intracellular potassium (K+) and influx of calcium (Ca2+) acutely exhaust local Na+/K + -ATPase pumps (23). If this profound depolarizing block propagates from the cortex down into the brainstem, it physically incapacitates the medullary networks, rendering the respiratory pacemakers and the ascending reticular activating system (ARAS) unresponsive to afferent stimuli (58). Electrophysiologically, this state of severe cerebral shutdown is captured as Post-ictal Generalized EEG Suppression (PGES). Historically, prolonged PGES (>50 s) was proposed as a potential biomarker for SUDEP risk, conceptualized as “bioelectric silence” indicative of an exhausted brain (20). However, a critical appraisal of contemporary literature reveals that the role of PGES as a standalone biomarker for SUDEP remains controversial. Studies have reported poor reproducibility between different clinical scorers and a high false-positive rate (56–59%) in automated detection algorithms, which contributes to high predictive uncertainty when relying on PGES alone (24). Furthermore, recent animal model data suggest the relationship between PGES duration and SUDEP risk is non-linear, with more severe brain injury paradoxically resulting in shorter suppression times (25). Consequently, PGES is increasingly viewed not as a definitive, independent risk marker, but rather as a surrogate indicator of brainstem dysfunction, a mediated step in a cascade that also involves impaired autonomic control.

Firstly, the methodological reproducibility of PGES is highly problematic. The clinical definition—typically relying on visual analysis to identify periods where EEG amplitude remains below 10 μV—is complicated by known technical challenges. According to the definition used in foundational studies, PGES is defined as the generalized absence of EEG activity >10 μV in amplitude, with allowance for muscle, movement, and respiratory interference in the immediate postictal period (within 30 s) (26). This inherent allowance for artifact makes precise measurement of PGES onset and duration notoriously difficult to standardize across different clinical scorers. The presence of post-ictal muscle artifact, wandering baselines, or respiratory artifact can indeed obscure the true cortical signal, contributing to high subjectivity across different epilepsy monitoring units.

Secondly, the predictive uncertainty of PGES has been starkly highlighted by recent large-scale, prospective multicenter cohorts. While early retrospective studies suggested a strong link between PGES duration and SUDEP, more rigorous analyses have largely failed to confirm this as a consistent independent risk factor. For instance, the REPO2MSE study (7)—a prospective, multi-center case–control study specifically designed to investigate SUDEP biomarkers—was unable to formally assess the association between PGES and SUDEP, as the low number of recorded focal-to-bilateral tonic–clonic seizures prevented this analysis. Furthermore, PGES is frequently observed in patients with GTCS who never experience SUDEP, and conversely, it may be absent in some witnessed SUDEP events (24). This suggests that PGES is more likely an epiphenomenon reflecting the severity and duration of the preceding motor convulsion (a concept supported by earlier literature on prior cohort characteristics) and the resulting brainstem dysfunction, respiratory failure, and cardiac instability, rather than a direct, independent trajectory toward death (20). Additionally, mechanisms leading to SUDEP are increasingly recognized as multi-system failures involving peri-ictal respiratory depression and cardiac instability, with PGES potentially representing only one downstream marker of this complex cascade (27).

Finally, a fundamental limitation of PGES lies in its spatial ambiguity. Standard scalp EEG primarily measures cortical suppression, but postmortem and experimental evidence suggests that PGES may be closely linked to brainstem dysfunction—specifically involving respiratory and cardiac regulatory centers—which is considered the final common pathway of SUDEP. While EEG alone cannot independently assess brainstem function, recent prospective studies have shown that peri-ictal apnoea (ictal central apnoea > 17 s and postictal central apnoea > 14 s) is a stronger independent predictor of SUDEP than EEG suppression alone. Furthermore, novel animal models demonstrate that PGES and/or spreading depolarizations can propagate from the forebrain to the brainstem, contributing directly to breathing abnormalities and death (60). Therefore, recognizing the limitations of PGES underscores a critical shift in the field: bioelectric silence alone is insufficient as a standalone biomarker. Whether the temporary loss of survival reflexes captured by PGES progresses to death or recovery depends heavily on concomitant cardiorespiratory function (peri-ictal apnoea, cardiac instability, and hypoxia) and environmental factors (such as solitary living and nighttime sleep) (28). This reinforces the necessity of moving away from isolated peri-ictal EEG markers toward integrated neuro-autonomic metrics, such as coupling EEG suppression with simultaneous SpO₂ monitoring and continuous ECG, to accurately gauge true physiological failure.

While SUDEP is classically conceptualized as a terminal post-ictal cascade triggered by a severe generalized convulsion, an overt clinical seizure is not an absolute prerequisite for the terminal event. The multisystem nature of this failure implies a heterogeneous process that does not strictly adhere to defined seizure phases. In patients harboring significant “dual pathology”—characterized by chronic autonomic dysregulation, impaired repolarization reserve, and structural remodeling of the ‘epileptic heart’—the baseline physiological reserve is critically eroded. Consequently, the multi-system abnormalities themselves may serve as the inciting event. In such highly vulnerable neuro-cardiac axes, a terminal arrhythmia or central apnea can spontaneously manifest interictally, or be precipitated by subclinical epileptiform discharges that fail to produce motor symptoms.

## 4 Risk factors

Risk stratification is the cornerstone of SUDEP prevention. Current epidemiological evidence has delineated a multidimensional landscape of predictors, which can be broadly categorized into modifiable clinical variables, fixed biological characteristics, and peri-ictal environmental circumstances.

### 4.1 Seizure severity and treatment resistance (major risk factors)

The frequency of generalized tonic–clonic seizures (GTCS) stands as the single most robust and consistent predictor of SUDEP. Rather than a strictly linear dose–response relationship, current evidence points toward a critical frequency threshold; while the risk is substantially amplified in patients experiencing frequent GTCS (typically defined as three or more per year), recent longitudinal data suggest that the risk profile may plateau beyond this threshold. This nuance implies that the cumulative burden of convulsive seizures drives systemic stress up to a point of physiological saturation, after which it precipitates terminal cardiorespiratory failure. Closely linked to this seizure burden is the presence of drug-resistant epilepsy (29). Non-adherence to antiseizure medications (ASMs) or the failure of polytherapy regimens serve as strong indicators of high-risk status. Crucially, achieving seizure freedom—even in historically refractory cases—remains the most effective strategy to negate this risk (30).

### 4.2 Peri-ictal circumstances and lack of supervision

The circumstances surrounding the terminal event provide vital mechanistic clues. The majority of SUDEP cases occur during sleep and are unwitnessed, highlighting the protective effect of nocturnal supervision. The absence of a bed partner or monitoring device often precludes timely intervention during the critical post-ictal phase. Furthermore, body position is a decisive factor. The “prone position” is documented in a significant majority of SUDEP cases (31). This position is hypothesized to mechanically compromise chest wall excursion and airway patency, exacerbating the central respiratory depression discussed in Section 3.1. When combined with seizure-induced immobility, the prone position creates a significant physical barrier to self-resuscitation.

### 4.3 Biological susceptibility and comorbidities

Beyond external circumstances and seizure characteristics, a patient’s intrinsic biological landscape plays a pivotal role in SUDEP susceptibility. The overlap between cardiac and neurological risk factors is increasingly understood through shared genetic vulnerabilities. A critical driver of this combined neuro-cardiac mechanism is the presence of pathogenic variants in genes that are co-expressed in both the brain and the heart.

These genetic channelopathies establish a profound dual pathology, simultaneously lowering the cerebral seizure threshold and intrinsically impairing cardiac repolarization reserve. For example, mutations in the *SCN5A* gene, which encodes the primary cardiac sodium channel Nav1.5, are classically associated with primary life-threatening inherited arrhythmias such as Long QT Syndrome (LQTS) type 3 and Brugada syndrome (“Mutations in SCN5A gene lead to long QT syndrome and progressive cardiac conduction defects,” n.d.). However, these mutations are now increasingly recognized for their expression in extra-cardiac tissues, directly linking them to altered cerebral excitability and an elevated risk for SUDEP. The functional overlap between primary arrhythmia syndromes and epilepsy suggests that in these patients, the heart is inherently primed to fail under the physiological stress of a seizure, representing a true state of arrhythmogenic susceptibility.

The genetic predispositions summarized in Table 1 serve as the molecular blueprint for this “perfect storm.” We prioritized genes such as *SCN1A*, *KCNH2*, *DEPDC5*, and notably *SCN5A* and *SCN8A*, as quintessential examples of this paradigm. Rather than operating through a single universal pathway, these genes illustrate the profound complexity of SUDEP susceptibility; each demonstrates how distinct mutations—ranging from ion channel dysfunction to structural mTOR hyperactivation—can critically impair both neurological and cardiovascular homeostasis, actively priming the neuro-cardiac axis for irreversible collapse.

**Table 1:** Key genetic drivers and the complexity of “dual pathology” in SUDEP.

Gene symbol	Primary protein/pathway	Neurological impact (brain)	Cardio-autonomic impact (heart/brainstem)	SUDEP complexity and specific pathomechanism
SCN1A	Voltage-gated Na + channel (Nav1.1)	Severe myoclonic epilepsy (Dravet syndrome); cortical hyperexcitability.	Altered cardiac electrophysiology; increased risk of fatal peri-ictal arrhythmias.	Synergistic vulnerability: indirect cardiac vulnerability secondary to severe seizure burden combined with intrinsic ion channel deficits in parasympathetic pathways.
SCN8A	Voltage-gated Na + channel (Nav1.6)	Early infantile epileptic encephalopathy (EIEE13); explosive epileptogenesis.	Marked bradycardia, perioral cyanosis, hypopnea, and repetitive ictal asystole.	Direct autonomic destabilization: gain-of-function (GOF) mutations directly disrupt both central seizure thresholds and cardiac pacemaking, causing highly specific, gene-driven dysautonomia.
SCN5A	Voltage-gated Na + channel (Nav1.5)	Altered cerebral excitability; elevated seizure susceptibility.	Long QT syndrome (LQTS) type 3; Brugada syndrome.	Tissue pleiotropy: primarily recognized as a cardiac channelopathy, but extra-cardiac expression directly links primary arrhythmogenic susceptibility to epilepsy risk.
KCNH2	Voltage-gated K + channel	Lowered seizure threshold; possible role in spreading depolarization.	Impaired repolarization reserve; LQTS phenotypes.	Shared repolarization defect: a true “dual pathology” where a single variant lowers the threshold for both cortical spreading depression and ventricular fibrillation under stress.
KCNT1	Na + −activated K + channel	Severe, early-onset seizures (DEEs).	Distinctive neuro-cardiac profiles.	Developmental arrest: mutations lead to profound, early-onset neurodevelopmental arrest intertwined with severe, baseline autonomic instability.
DEPDC5	mTOR signaling regulator	Focal cortical dysplasia (FCD); increased neuronal size and excitability.	Structural remodeling of CAN regions; cardiac hypertrophy and dysfunction.	Structural remodeling: shifts the mechanism from purely functional (ion channel) to structural, driving physical malformations in both brain networks and the myocardium.

**Figure 2:**
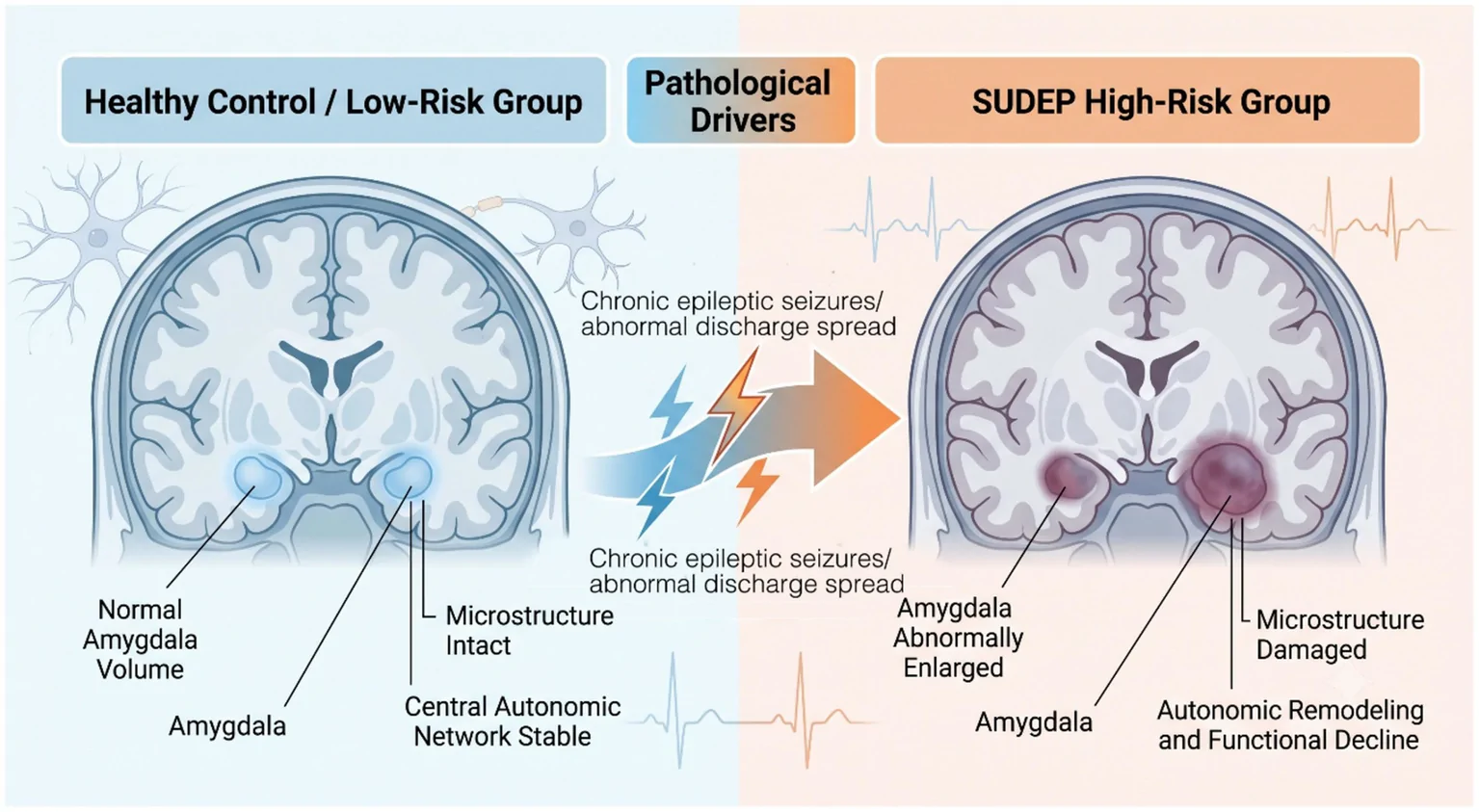
Structural and microstructural neuroimaging biomarkers in SUDEP.

These genetic risks are often compounded by structural cerebral anomalies (32). Building upon the functional role of the amygdala in suppressing respiratory drive during seizures, recent neuroimaging advancements have highlighted its structural vulnerability as a critical indicator of SUDEP susceptibility. Notably, Legouhy et al. (33) demonstrated that patients with focal epilepsy exhibiting varied levels of SUDEP risk possess distinct volumetric and microstructural abnormalities within the amygdala (Figure 2). These structural alterations suggest that chronic epileptic networks can drive the physical remodeling of crucial central autonomic hubs, thereby progressively eroding the physiological reserve required to survive a peri-ictal autonomic storm. Consequently, integrating such targeted microstructural and volumetric amygdala metrics into multimodal assessments provides a highly valuable neuroimaging biomarker. This enhances patient-level digital phenotyping and offers a non-invasive, objective tool for identifying individuals at the highest risk of fatal respiratory failure before a terminal event occurs.

**Figure 3:**
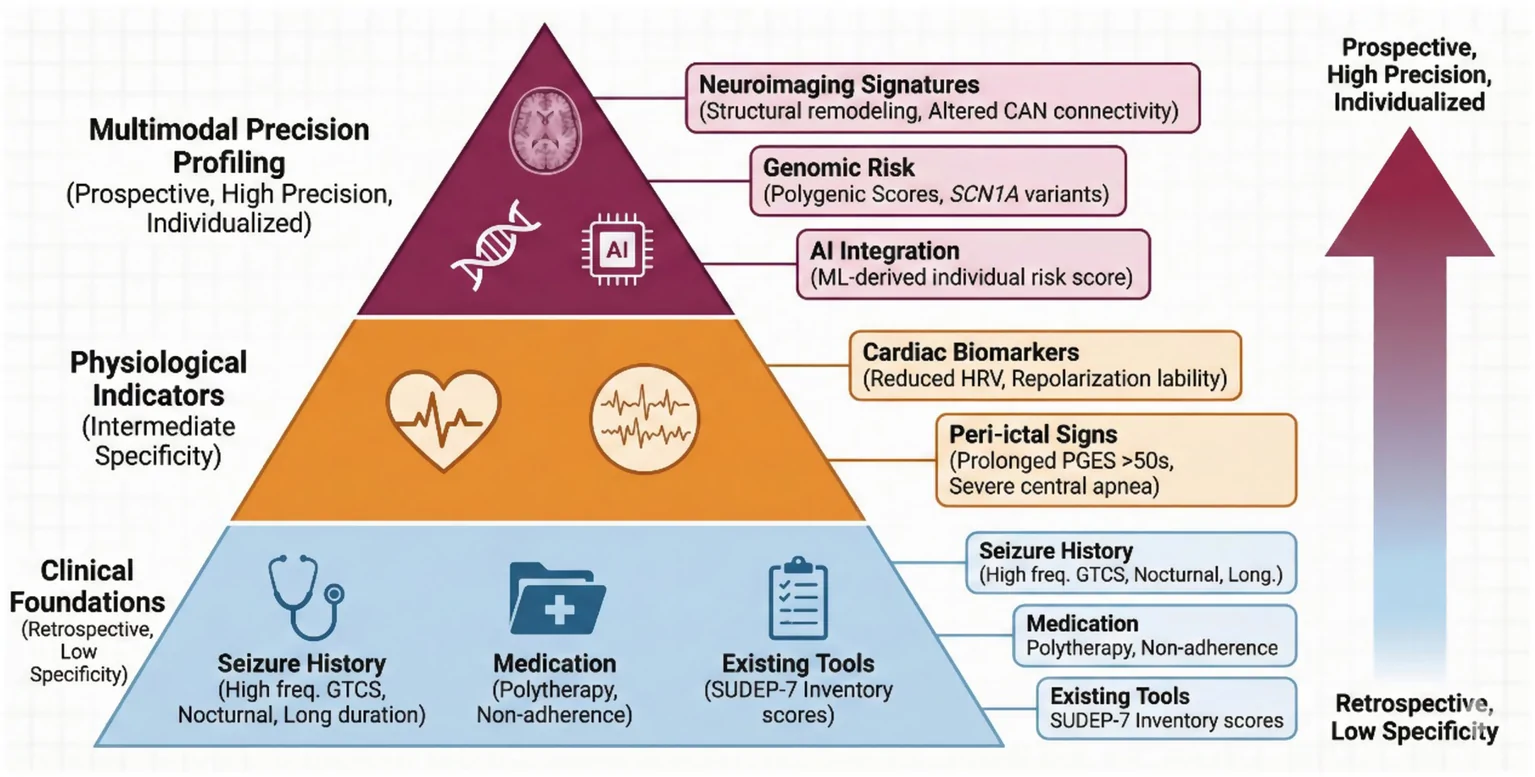
The evolving pyramid of SUDEP risk stratification: Moving toward precision medicine.

Collectively, these clinical, environmental, and biological factors paint a complex picture of SUDEP susceptibility. In the context of our proposed risk assessment model, these variables constitute the “Clinical Foundations” at the base of the risk stratification pyramid (Figure 3). While these foundational markers are essential for identifying high-risk cohorts, they represent the starting point of a diagnostic journey that must ascend toward more dynamic and individualized physiological monitoring.

### 4.4 Developmental and epileptic encephalopathies (DEEs): genotype-specific risks and autonomic vulnerability

Developmental and epileptic encephalopathies (DEEs) represent a highly vulnerable cohort where the cumulative burden of severe, intractable seizures intersects with profound developmental and neurological deficits. In these monogenic neurodevelopmental disorders, the abnormal neurodevelopment is intrinsically linked to both the underlying genetic defect and the severe epileptic activity itself. This population exhibits a markedly elevated risk for Sudden Unexpected Death in Epilepsy (SUDEP), emphasizing that SUDEP vulnerability is not universally uniform but is deeply influenced by specific genotype–phenotype correlations.

The severe epilepsy phenotypes characteristic of DEEs, such as frequent, drug-resistant generalized tonic–clonic seizures, nocturnal clustering, and prolonged convulsive events, serve as major clinical drivers of the terminal cardiorespiratory cascade (34). Furthermore, many children with DEEs present with significant comorbidities, including profound hypotonia, impaired airway clearance, and baseline autonomic dysregulation (dysautonomia), which severely compromise their ability to mount physiological arousal reflexes or mechanically recover from post-ictal prone positioning (35). Crucially, emerging evidence indicates that specific genotypes within the DEE spectrum carry distinct, intrinsic risks for autonomic collapse, further illustrating the concept of “dual pathology” across the neuro-cardiac axis. While *SCN1A* loss-of-function variants (Dravet syndrome) are well-established paradigms of this overlap, the expanding genomic landscape of DEEs highlights other critical culprits (36). Most notably, pathogenic gain-of-function (GOF) variants in the *SCN8A* gene (encoding the Nav1.6 voltage-gated sodium channel) result in a severe DEE (EIEE13) that carries an exceptionally high early mortality and SUDEP rate, estimated at approximately 10% (37).

The pathophysiology of *SCN8A*-related DEE exemplifies a profound, gene-specific failure of autonomic homeostasis. Seizures in *SCN8A* patients are frequently characterized by early, stereotyped autonomic manifestations, including marked bradycardia, perioral cyanosis, hypopnea, and even repetitive ictal asystole requiring cardiopulmonary resuscitation. Because the Nav1.6 channel regulates neuronal excitability across both central pathways and autonomic circuits, the GOF mutation not only drives severe epileptogenesis but also directly destabilizes cardiac rhythm during the peri-ictal phase (34).

Similarly, other DEE-associated genes, such as *KCNT1* (encoding a sodium-activated potassium channel), cause severe, early-onset seizures with distinctive neuro-cardiac profiles (38). The recognition of these genotype-specific risk profiles holds profound translational relevance for SUDEP prevention. It mandates a shift towards precision therapies; for instance, leveraging sodium channel blockers (SCBs) may be highly effective and protective in *SCN8A* GOF encephalopathies, whereas they are typically contraindicated in *SCN1A* loss-of-function cases. Furthermore, patients harboring high-risk DEE variants with known autonomic instability (e.g., *SCN8A*) represent the prime candidates for aggressive, early deployment of nocturnal monitoring devices and potentially, in life-threatening scenarios of ictal asystole, the consideration of cardiac pacemaker implantation.

By integrating genotype, seizure severity, and baseline autonomic dysfunction, the evolving understanding of DEEs provides a critical window into the molecular and physiological mechanisms that orchestrate the perfect storm of SUDEP.

## 5 Biomarkers and risk assessment models: distinguishing established, translational, and speculative approaches

Contemporary risk assessment in SUDEP is undergoing a critical evolution. To accurately interpret this landscape, it is essential to delineate validated clinical practices from emerging research. This evolution is conceptualized through a hierarchical pyramid, moving from established clinical foundations to promising physiological indicators, and finally to speculative precision profiling (Figure 3).

### 5.1 Established evidence: clinical surrogates and their limitations

At the foundation of validated risk assessment are established clinical scoring systems, most notably the SUDEP-7 Inventory (39). This tool relies on robust, established evidence such as GTCS frequency and epilepsy duration. While SUDEP-7 effectively validates the cumulative burden of the disease, it historically functioned primarily as a static historical ledger. However, in modern practice, this inventory directly benefits the individual patient by providing a standardized, easily calculable baseline score that empowers clinicians to quickly flag individuals at elevated risk during routine outpatient visits (40). This proactive identification is crucial for implementation into current monitoring paradigms; an elevated SUDEP-7 score provides the clinical justification to transition a patient from standard care to more intensive, continuous monitoring, such as ambulatory step-down telemetry or home-based nocturnal surveillance. As highlighted by Ochoa-Urrea et al. (6), reliance on historical clinical surrogates alone yields low positive predictive values for forecasting individual terminal events, necessitating the investigation of dynamic physiological metrics.

### 5.2 Promising translational biomarkers: autonomic and polysomnographic indicators

To address the limitations of static inventories, research has pivoted toward promising translational biomarkers that continuously capture autonomic integrity. Interictal Heart Rate Variability (HRV) is emerging as a robust, non-invasive proxy for autonomic tone (41). High-risk patients exhibit a progressive decline in specific HRV domains (e.g., RMSSD, HF power) and an altered Low Frequency/High Frequency (LF/HF) ratio, reflecting a state of chronic parasympathetic withdrawal and sympathetic dominance (40, 42).

Crucially, dynamic risk stratification increasingly relies on elaborating neuro-autonomic coupling to understand the bidirectional brain-heart interaction. Advanced analytical metrics, such as the cross-correlation between the continuous HRV signal and the amplitude envelope in particular EEG frequency bands (e.g., theta and delta bands during the post-ictal period), allow clinicians to dynamically quantify the breakdown in communication between cortical activity and brainstem autonomic regulatory centers. Concurrently, dynamic QT interval dispersion and T-wave alternans are being investigated as translational markers of an electrical substrate vulnerable to lethal ventricular arrhythmias.

Building upon these autonomic markers, dynamic risk stratification increasingly incorporates high-resolution polysomnography (PSG). Original case–control studies have identified highly specific predictive signatures: profound peri-ictal hypoxemia (SpO2 < 75%) coupled with prolonged central apnea occurring specifically during NREM sleep (43). Integrating these translational markers (HRV coupling and PSG signatures) into current monitoring paradigms—such as utilizing multi-day Holter monitors or at-home level-II PSG screening devices—provides immense benefit to the individual patient. It identifies subclinical physiological exhaustion, guiding clinicians to initiate targeted interventions like adjusting antiseizure medications, prescribing nocturnal oxygen supplementation, or prioritizing the patient for resective surgery evaluation.

### 5.3 Speculative and future-oriented concepts: artificial intelligence and digital phenotyping

**Figure 4:**
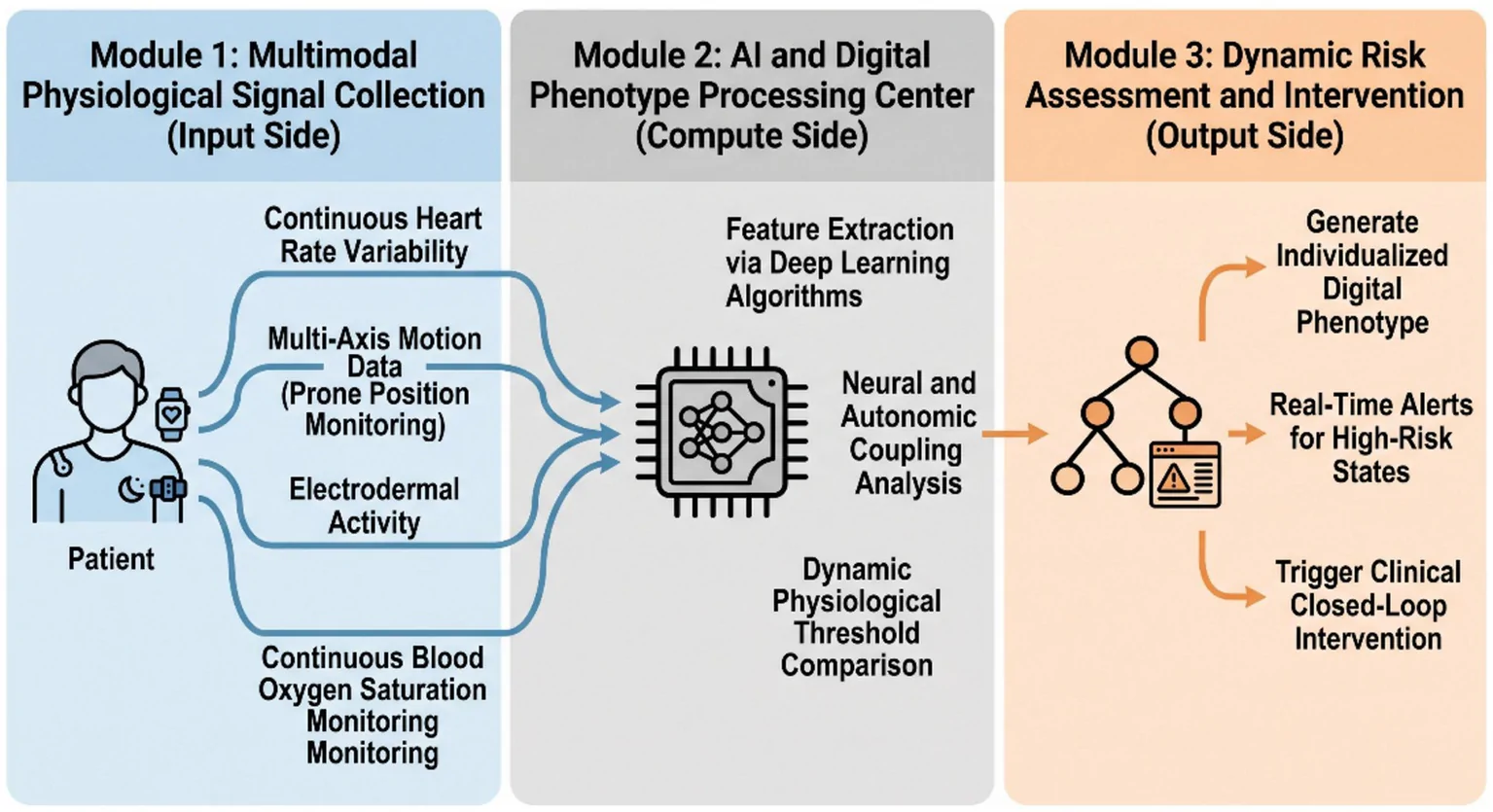
Multimodal wearable platforms and digital phenotyping for SUDEP prevention.

The integration of wearable devices and Artificial Intelligence (AI) into epilepsy care represents a major technological advancement. Active secondary prevention currently relies on clinically validated, FDA-cleared wearable technologies. Moving beyond rudimentary bed partner alerts and basic alarms, contemporary platforms are capable of highly granular physiological monitoring. For instance, NightWatch+ is an FDA-cleared wireless device that monitors nocturnal seizures; crucially, it already detects continuous heart rate and multi-axis motion, which is highly relevant to assessing the mechanical risk of prone positioning during a seizure (Figure 4).

These devices utilize established machine learning algorithms to analyze peripheral autonomic signals, specifically combining electrodermal activity (EDA) with multi-axis accelerometry (ACM) to distinguish severe generalized tonic–clonic seizures (GTCS) from normal nocturnal movements. Deployed in both epilepsy monitoring units and outpatient settings, these devices serve a focused clinical purpose: to trigger automated caregiver alerts and facilitate timely intervention during the critical post-ictal window.

Moving beyond overt seizure detection, the concept of predicting individual SUDEP trajectories through “digital phenotyping” is rapidly expanding. Cutting-edge AI architectures and deep learning frameworks are being utilized to process diverse, simultaneous data streams. These models integrate wearable EEG, continuous ECG, respiratory effort, and SpO2 to dynamically quantify the breakdown in the aforementioned neuro-autonomic coupling (57). By identifying subclinical shifts in LF/HF ratios and SpO2 variability long before a fatal event occurs, digital phenotyping aims to generate a personalized, predictive risk score for continuous patient management.

Despite the rapid advancement of digital health tools, several critical limitations impede their universal real-world implementation. A primary clinical challenge is the elevated rate of false alarms generated by available wearable sensors, which frequently leads to caregiver “alarm fatigue” and subsequent non-compliance. Furthermore, AI-driven predictive models are severely impacted by dataset heterogeneity. Algorithms trained on highly controlled, pristine data from Epilepsy Monitoring Units (EMUs) frequently lack external validation when applied to diverse, ambulatory patient populations. Ultimately, standardizing multimodal data collection and improving algorithm specificity in uncontrolled environments are essential prerequisites for transitioning these AI and digital phenotyping technologies from experimental milestones to robust clinical standards.

## 6 Prevention strategies: from risk stratification to personalized interventions

The preventability of Sudden Unexpected Death in Epilepsy (SUDEP) hinges on a fundamental paradigm shift from generalized risk mitigation to precision medicine. While the preceding sections delineated how clinical, genomic, and physiological biomarkers identify an individual’s risk profile, effective clinical care requires directly mapping these specific vulnerabilities to tailored therapeutic strategies. Rather than employing a uniform approach, prevention must be individualized across different categories of risk.

### 6.1 Precision management of genetic and developmental epileptic encephalopathies (DEEs)

For patients harboring specific genetic vulnerabilities, primary prevention relies heavily on genotype-driven medication management and targeted supervision. As highlighted in Section 4.4, the concept of “dual pathology” dictates that treatments must address both cortical hyperexcitability and inherent autonomic instability. For instance, patients with *SCN8A* gain-of-function variants—who exhibit severe dysautonomia and ictal asystole—may benefit from the targeted use of sodium channel blockers to stabilize both neural and cardiac pathways. Conversely, these same agents are largely contraindicated in patients with *SCN1A* loss-of-function variants (Dravet syndrome). Furthermore, for individuals with established DEEs featuring profound hypotonia and impaired physical arousal reflexes, personalized care mandates the aggressive early deployment of nocturnal monitoring devices. In extreme phenotypes presenting with documented, recurrent ictal asystole, proactive multidisciplinary evaluation for cardiac pacemaker implantation becomes a highly specific, individualized preventative measure.

### 6.2 Targeted interventions for drug-resistant focal epilepsy (DRE)

In patients with DRE and structurally defined epileptogenic zones, the personalized approach prioritizes halting the cumulative structural erosion of the “epileptic heart.” Because the frequency of generalized tonic–clonic seizures (GTCS) exhibits a linear dose–response relationship with mortality risk, achieving absolute seizure freedom remains the gold standard. Resective surgery or minimally invasive laser thermal ablation should be expedited for eligible candidates, as removing the seizure focus fundamentally negates the downstream terminal cascade (44).

For individuals with non-surgical DRE, early and aggressive medical escalation is mandatory, and the choice of neuromodulation should be carefully individualized. Vagus Nerve Stimulation (VNS), for example, offers targeted benefits for patients exhibiting chronic parasympathetic withdrawal. Recent evidence demonstrating resting-state functional connectivity changes following microburst VNS therapy suggests it exerts a dual protective effect by actively modulating autonomic networks to buffer against peri-ictal cardiac instability, distinct from its primary anticonvulsant properties, leading to a significant long-term reduction in SUDEP rates (45). Furthermore, Responsive Neurostimulation (RNS) systems provide a highly personalized approach for patients with eloquent focal onset zones. Long-term clinical trial data indicate that continuous, closed-loop RNS significantly reduces the occurrence of SUDEP compared to baseline rates in treatment-resistant populations, likely by persistently preventing seizure propagation into the central autonomic networks (46).

### 6.3 Phenotype-specific technological vigilance

The deployment of wearable technologies must be aligned with the patient’s specific risk category and “digital phenotype” (47). Solitary patients or those with a high SUDEP-7 score driven by nocturnal GTCS require multimodal devices that integrate accelerometry and electrodermal activity (EDA) to detect convulsive events and trigger immediate caregiver intervention (48). However, for individuals whose dynamic biomarker profiling (e.g., polysomnography) reveals a severe vulnerability to peri-ictal central apnea and profound hypoxemia, standard motion-based alarms are insufficient. In these specific cases, individualized surveillance protocols should strictly incorporate continuous SpO2 monitoring and evaluate the necessity of nocturnal oxygen supplementation to directly counteract the respiratory component of the “perfect storm” (49).

### 6.4 The frontier of secondary prevention: phenotype-specific resuscitation

Looking toward a longer-term horizon, secondary prevention aims to develop intelligent, closed-loop systems capable of autonomous intervention tailored to the patient’s primary failure mode. Upon detecting critical post-ictal central apnea, an idealized system would automatically initiate a tiered therapeutic response. For a patient phenotyped with primary respiratory arrest, this could involve targeted diaphragmatic electrical stimulation to maintain ventilation (50).

Simultaneously, pharmacological resuscitation strategies—such as the automated delivery of adenosine A1 or A2A receptor antagonists—aim to counteract the surge of inhibitory neuromodulators that paralyze medullary pacemakers. Crucially, translating these interventions from concept to personalized clinical application requires rigorous validation. Because SUDEP involves an integrated failure of the whole neuro-cardiac axis, the efficacy and safety of these tailored interventions must be strictly evaluated using *in vivo* animal models that accurately replicate systemic cardiorespiratory collapse, as *in vitro* cellular studies cannot capture the complex, multi-system dynamics of the terminal cascade.

## 7 Research gaps and the path toward clinical implementation

Despite significant advancements in characterizing the “perfect storm,” several critical gaps preclude the universal implementation of SUDEP prevention protocols. First, the field lacks a unified, validated threshold for physiological markers; while Heart Rate Variability (HRV) and Post-ictal Generalized EEG Suppression (PGES) are robust indicators, their predictive granularity remains insufficient for real-time individual forecasting. To bridge this gap, future research must establish standardized, multimodal composite metrics. For HRV, moving beyond isolated time-domain parameters to continuous frequency-domain analyses (such as dynamic LF/HF ratio tracking) is essential. For PGES, isolated duration thresholds must be replaced by indices that strictly couple EEG suppression with simultaneous SpO2 desaturation durations and ECG repolarization abnormalities. The clinical implementation of these metrics requires large-scale, prospective multicenter registries that utilize standardized machine learning algorithms to analyze data from ambulatory wearable sensors, thereby establishing reliable, individualized baseline thresholds rather than relying on generalized population averages.

Furthermore, the dual pathology of genetic variants, such as DEPDC5, highlights a complex intersection between neuronal excitability and cardiomyocyte dysfunction that current clinical inventories often fail to capture. Addressing this gap requires a paradigm shift toward proactive genomic medicine. We suggest the routine integration of comprehensive genetic screening for known SUDEP-associated variants (e.g., *SCN1A*, *SCN8A*, *DEPDC5*) in all patients presenting with treatment-resistant or early-onset epilepsy. For susceptible patients identified through this screening, clinical care pathways must mandate long-term remote monitoring. Implementing advanced, continuous surveillance technologies, such as implantable loop recorders or highly specific multimodal wearables in these genetically vulnerable cohorts is crucial to capture the transient, subclinical cardiopulmonary dysfunctions that precede a terminal collapse.

A major controversy persists regarding the “unified hypothesis” of SUDEP, with some researchers arguing that the multi-system cascade is too heterogeneous to be reduced to a single terminal pathway (51). Furthermore, this controversy extends to the profound diagnostic ambiguity between SUDEP and Sudden Cardiac Death (SCD) in the epilepsy population. As patients endure chronic epilepsy, the neuro-cardiac axis often undergoes cumulative cardiac remodeling, frequently manifesting as underlying cardiomyopathies and cardiac fibrosis which heavily facilitates lethal arrhythmias (52, 53). Complicating this diagnostic landscape is the pharmacological burden of antiseizure medications (ASMs). While chronic ASM polytherapy is a recognized epidemiological risk marker, certain agents (e.g., specific sodium channel blockers) may possess intrinsic proarrhythmic properties or exacerbate underlying repolarization vulnerabilities. Consequently, the iatrogenic impact of these medications may contribute more prominently to interictal SCD than to classic seizure-induced SUDEP, further blurring the lines between neurological and primary cardiac mortality. Although epidemiologic patterns suggest differing clinical profiles—SCD in epilepsy tends to peak at a later age and is more likely to occur interictally, whereas classic SUDEP is strongly associated with the post-ictal phase in younger demographics—the relative contribution of primary cardiac etiologies versus seizure-induced multisystem collapse remains highly controversial (54). Ultimately, prioritizing the development of AI-driven models that can dynamically weigh these disparate factors within a “digital phenotype” is necessary to move beyond retrospective risk identification toward proactive, personalized intervention.

## 8 Conclusion and future perspectives

Sudden Unexpected Death in Epilepsy (SUDEP) serves as a critical reminder that epilepsy is a systemic disorder with profound cardiorespiratory implications, rather than a condition confined solely to cerebral hyperexcitability. This review complements current clinical summaries by emphasizing a translational framework centered on “dual pathology” and digital innovation. We highlight that the multi-system collapse is frequently primed by combined neuro-cardiac vulnerabilities, which standard clinical inventories may not capture dynamically. Recognizing this complex pathophysiology suggests that the future of SUDEP prevention will benefit from a multidisciplinary convergence of genomics, artificial intelligence, and biomedical engineering.

The translation of this knowledge into clinical practice requires a cultural shift in how risk is managed. The historical reluctance to discuss SUDEP due to concerns about inducing patient anxiety is increasingly recognized as counterproductive (55). Instead, risk stratification should become an integral component of routine epilepsy care, akin to monitoring cardiovascular risk factors.

Looking to the horizon, the convergence of precision medicine and digital health heralds a new phase of clinical management. The continued validation of digital phenotypes—integrating genomic, physiological, and lifestyle data—aims to predict individual vulnerability with higher fidelity. Concurrently, the ongoing research into closed-loop neurostimulation and automated intervention systems offers the theoretical potential to intervene before a seizure evolves into a terminal cascade. While significant translational challenges remain, the rigorous application of these scientific and engineering advancements outlines a clear path toward making SUDEP an actively preventable clinical entity.
